# Exploring the psychopathological profile of fibromyalgia: insights from the personality assessment inventory and its association with disease impact

**DOI:** 10.3389/fpsyg.2024.1418644

**Published:** 2024-09-12

**Authors:** Andrea Doreste, Jesus Pujol, Eva Penelo, Víctor Pérez, Laura Blanco-Hinojo, Gerard Martínez-Vilavella, Helena Pardina-Torner, Fabiola Ojeda, Jordi Monfort, Joan Deus

**Affiliations:** ^1^Department of Clinical and Health Psychology, Autonomous University of Barcelona, Barcelona, Spain; ^2^MRI Research Unit, Department of Radiology, Hospital del Mar, Barcelona, Spain; ^3^Instituto de Salud Carlos III, Centro de Investigación Biomédica en Red de Salud Mental (CIBERSAM G21), Barcelona, Spain; ^4^Department of Psychobiology and Methodology of Health Sciences, Autonomous University of Barcelona, Barcelona, Spain; ^5^Neurociences Research Unit, IMIM-Institut Hospital del Mar d’Investigacions Mèdiques, Barcelona, Spain; ^6^Department of Experimental and Health Sciences, Pompeu Fabra University, Barcelona, Spain; ^7^Cognition and Brain Plasticity Unit, Bellvitge Biomedical Research Institute–IDIBELL, Barcelona, Spain; ^8^Department of Rheumatology, Hospital del Mar, Barcelona, Spain

**Keywords:** disease impact, fibromyalgia, fibromyalgia impact questionnaire (FIQ), psychopathology, personality assessment inventory (PAI)

## Abstract

**Background:**

Fibromyalgia (FM) is a complex rheumatic disorder characterized by chronic nociplastic pain and central sensitization. Psychopathological conditions can influence FM symptoms, which worsen their condition. However, not all patients with FM have psychopathological disorders, indicating a heterogeneous population.

**Objective:**

To investigate the psychopathological profile and personality disorders in patients with FM and its relationship impact on this disease.

**Methods:**

An observational and cross-sectional comparative study was conducted with a sample of 90 women, mean age 48.7 years (SD = 8.12), from Hospital del Mar, Barcelona. The Personality Assessment Inventory (PAI) and the Fibromyalgia Impact Questionnaire (FIQ) were used for assessment.

**Results:**

FM patients predominantly exhibited psychopathological profiles resembling affective disorders (37.7%) and Cluster C personality disorders (58.8%). The severity of FM’s impact was related to affective disorder symptoms, hypervigilance, derealization, somatization, and Cluster B personality disorder (emotional instability). Different rheumatic symptoms correlated with specific psychopathological patterns. Increased somatic symptoms on the FIQ were related to an unstable and dependent personality, while heightened emotional symptoms on the FIQ were associated with avoidance, borderline traits, and passive-aggressive reactions.

**Conclusion:**

Recognizing psychopathological aspects is crucial for managing FM. The PAI is a valuable tool for establishing its psychopathological multidimensional profile, which predominantly shows an affective spectrum conditions and comorbid Cluster C personality disorder, exacerbating the disease’s impact.

## 1 Introduction

Fibromyalgia (FM) is a common rheumatic disease, mainly affecting women, with 2–4% prevalence in the general population (Bair and Krebs, 2020; Sarzi-Puttini et al., 2020). It falls under the category of Central Sensitization (CS) syndromes (De Tommaso et al., 2022), characterized by chronic nociplastic pain (altered nociception despite no clear evidence of tissue damage or inflammation) (Sarzi-Puttini et al., 2020; De Tommaso et al., 2022). However, its subjective symptomatology poses challenges for objective assessment using conventional medical tests (Sarzi-Puttini et al., 2020; Pujol et al., 2019), although neuroimaging studies have revealed Central nervous system (CNS) dysfunction in processing both nociceptive and non-nociceptive inputs. At present, diagnosing FM relies on subjective clinical symptoms, primarily widespread musculoskeletal pain, often accompanied by fatigue, unrefreshing sleep, other somatic disturbances, cognitive dysfunction, and mental health problems (Bair and Krebs, 2020; Sarzi-Puttini et al., 2020). The etiology of FM is intricate, considered multifactorial and idiopathic (Bair and Krebs, 2020; Sarzi-Puttini et al., 2020; De Tommaso et al., 2022), with psychological and psychopathological factors potentially modulating FM’s complex array of symptoms (García-Fontanals et al., 2016; García-Fontanals et al., 2017; Karlin et al., 2005) or increasing vulnerability according to the diathesis-stress model (González et al., 2021).

Patients with FM often experience elevated psychological distress, including anxiety and depression, functional limitations, maladaptive thinking patterns, and ineffective coping strategies, all contributing to heightened pain perception (García-Fontanals et al., 2017; González et al., 2021; Bucourt et al., 2021). Nevertheless, research suggests that individuals diagnosed with FM form a diverse group based on their rheumatic symptom (Keller et al., 2011; Torres et al., 2013), personality traits (González et al., 2021; Torres et al., 2013), and psychological distress or psychopathological profiles (García-Fontanals et al., 2017). While not all patients with FM show a psychopathological disorder (García-Fontanals et al., 2017; Gálvez-Sánchez et al., 2019), recent systematic reviews reveal a high prevalence of psychiatric comorbidities (13–80%) (Gálvez-Sánchez et al., 2019). Major depressive disorder (19–65%) (García-Fontanals et al., 2017; Kleykamp et al., 2021; Løge-Hagen et al., 2019) and persistent depression or dysthymia (50–53%) (García-Fontanals et al., 2017; Kleykamp et al., 2021) are the most prevalent. While FM is no longer considered a somatic mental disorder (somatoform), both biological and psychosocial factors play pivotal roles in exacerbating and perpetuating somatic symptoms in these patients (Häuser and Henningsen, 2014).

Most current research indicates that the prevalence of personality disorders (PDs) diagnosed in FM patients is significantly higher than in the general population. However, the data regarding the specific subtypes of PDs associated with FM remains contentious.Specific personality traits have been identified in FM, proposing Type D Personality, characterized by greater negative emotionality and social introversion, which is present in 56.5% of patients (van Middendorp et al., 2016; Gokcen et al., 2022). Moreover, personality disorders (PDs) are common comorbidities (García-Fontanals et al., 2016; Kleykamp et al., 2021; Attademo and Bernardini, 2018; Gumà-Uriel et al., 2016), with a broad prevalence range of 8.7–96.7% (Attademo and Bernardini, 2018) with an estimated mean of 19.3% (Kleykamp et al., 2021). Most studies emphasize that the predominant PDs diagnosed in FM patients belong to Cluster C disorders, according to the Diagnostic and statistical manual of mental disorders (DSM) V/5th ed. (Gumà-Uriel et al., 2016), with high levels of anxiety and avoidance behavior (García-Fontanals et al., 2016; Kleykamp et al., 2021; Attademo and Bernardini, 2018; Gumà-Uriel et al., 2016). Specifically, the most prevalent diagnoses are obsessive-compulsive and avoidant personality disorder (García-Fontanals et al., 2016; Attademo and Bernardini, 2018; Gumà-Uriel et al., 2016), with borderline and histrionic personality disorder in Cluster B being less prevalent (Attademo and Bernardini, 2018). Additionally, some studies report high rates of both cluster B and cluster C personality disorders (PDs). However, there is no data on the impact of cluster A PDs, despite some reports of co-occurrence between fibromyalgia (FM) and paranoid or schizoid PDs (Romanov et al., 2023).

Despite the need to grasp FM’s psychopathological aspects, many FM studies have used limited psychological distress assessments (Karlin et al., 2005), overlooking multidimensional instruments for defining the psychopathological profile and personality disorders according to DSM-5 criteria (Gumà-Uriel et al., 2016; Novo et al., 2017). Psychopathological exploration of FM should be multidimensional (Karlin et al., 2005), with the Minnesota Multiphasic Personality Inventory (MMPI) (González et al., 2021; Novo et al., 2017), the Millon Clinical Multiaxial Personality Inventory (MCMI) (García-Fontanals et al., 2017; López-Ruíz et al., 2019) or the symptom checklist-90-revised (SCL-90-R) (Fillingim et al., 2020) most commonly utilized. Surprisingly, the Personality Assessment Inventory (PAI), which effectively defines psychopathological profiles and personality disorders based on DSM-5 criteria (Karlin et al., 2005; Romanov et al., 2023), has been underused in chronic pain patients (Karlin et al., 2005). The PAI assess several constructs with satisfactory psychometric properties, offering comprehensive coverage of personality and psychopathology phenomena relevant to the chronic pain population, such as FM patients, often not adequately represented by other multidimensional assessments (Karlin et al., 2005). Specifically, the PAI’s endorsement of somatic complaints, highly prevalent in FM, does not artificially inflate other clinical-subclinical scale scores, a limitation of other multidimensional tools (Karlin et al., 2005).

Hence, the PAI appears valuable for uncovering a range of psychological and personality issues in chronic pain patients, particularly those with FM. To date, there are no published studies on PAI use exclusively with FM patients (Sarzi-Puttini et al., 2020; Burneo-Garcés et al., 2020). Therefore, this study aims to assess the clinical utility of the PAI in diagnosing psychopathological profiles and personality disorders in FM patients. It also explores their association with illness severity and impact. Ultimately, this research seeks a comprehensive grasp of psychological factors in FM to pinpoint intervention and treatment opportunities.

## 2 Methods

This study adhered to the principles outlined in the Declaration of Helsinki and received approval from the Ethical Committees: Parc de Salut Mar of Barcelona (reference 6,932/I) and the Commission on Ethics in Animal and Human Experimentation (CEEAH) at the Autonomous University of Barcelona (UAB) (reference 6,496). Written informed consent was obtained from all patients.

### 2.1 Eligibility criteria

The study involved female aged 18 to 65 with a clinical FM diagnosis based on American College of Rheumatology criteria (Wolfe et al., 2010). Inclusion criteria comprised stable pharmacological treatment, comprehension of study requirements, and a commitment to compliance. Exclusions encompassed other pain-explaining disorders, inflammatory or rheumatic diseases, severe or unstable medical, endocrine or neurological conditions, neuropathic pain history, acute-psychotic disorder, drug abuse, and PAI validity scale scores that invalidating the interpretation.

### 2.2 Participants

Patients were recruited at the Fibromyalgia Unit of the Hospital del Mar of Barcelona by senior rheumatologists (FO or JM) and a senior psychologist (JD) from January 2021 to June 2022. Out of 136 female patients with a clinical FM diagnosis, 110 were assessed for eligibility following consecutive order during clinical visits. Eleven patients failed to meet study criteria, and nine declined participations, leaving a final sample of 90 females who completed the PAI. Data from 20 patients who did not adequately complete the Fibromyalgia Impact Questionnaire (FIQ) were excluded from some PAI analyses. Relevant sociodemographic and clinical characteristics are detailed in Table 1.

**Table 1:** Clinical and sociodemographic characteristics of the female sample (*N* = 90).

Age (years) M (SD)	48.73 (8.12)
Fibromyalgia tender points (0–18) M (SD)	17.01 (2.22)
Years from FM diagnostic M (SD)	6.81 (6.45)
Level of studies (%)	
Primary studies	8.6
Secondary studies	12.3
Bachelor	17.3
Professional studies	30.9
University	30.9
Fibromyalgia associated symptoms (0–100)* M (SD)	
Morning tiredness	76.6 (21.79)
Unrefreshed sleep	74.78 (20.25)
Fragmented sleep	62.53 (32.36)
Fatigue	79 (14.52)
Morning stiffness	72.02 (23.9)
Stiffness after resting	59.51 (27.9)
Subjective swelling	51.52 (32.89)
Paraesthesia’s	60.10 (27.35)
Headache	59.26 (32.1)
Symptoms of irritable bowel	42.84 (37.8)
Depression symptoms	57.86 (30.94)
Anxiety symptoms	61.42 (34.67)
Attention and concentration (0–100)* M (SD)	65.31 (26.07)
Memory complaints (0–100)* M (SD)	64.94 (25.68)
FIQ**: global score (0–100) M (SD)	66.81 (13.67)
Physical dysfunction (0–10) M (SD)	5.84 (2.24)
General discomfort (0–10) M (SD)	8.01 (2.54)
Sick leave caused by FM (0–10) M (SD)	4.36 (3.48)
Pain at work (0–10) M (SD)	6.99 (1.95)
Pain (0–10) M (SD)	7.14 (1.64)
Fatigue (0–10) M (SD)	7.84 (1.36)
Morning tiredness (0–10) M (SD)	7.46 (2.08)
Stiffness (0–10) M (SD)	6.8 (2.46)
Anxiety (0–10) M (SD)	6.6 (2.65)
Depression (0–10) M (SD)	5.54 (2.89)
Stable medication regime (%)	
Analgesic (NSAIDs and/or Opioids)	69.2
Anti-inflammatory	62.8
Antidepressant	73.1
Type of antidepressant	
ISRS	30.8
Dual	17.9
Tricyclic	25.6
Benzodiazepine	34.6
Type of benzodiazepine	
Short life	15.4
Medium life	3.8
Long life	15.4

*Subjective complaints assessment according to a visual analog scale (VAS), maximum score 100.

### 2.3 Study design and procedure

We used a non-randomized, purposeful sampling method to include all eligible participants from the study population. This was an observational, cross-sectional study. Female patients initially attended their regular rheumatology appointment (FO and JM). After thorough screening for inclusion/exclusion criteria and willingness to participate, they were enrolled. Psychological assessments, conducted by another clinical psychologist (AD), occurred within the same week and lasted up to an hour and a half to prevent response fatigue.

### 2.4 Instruments

**Figure 1:**
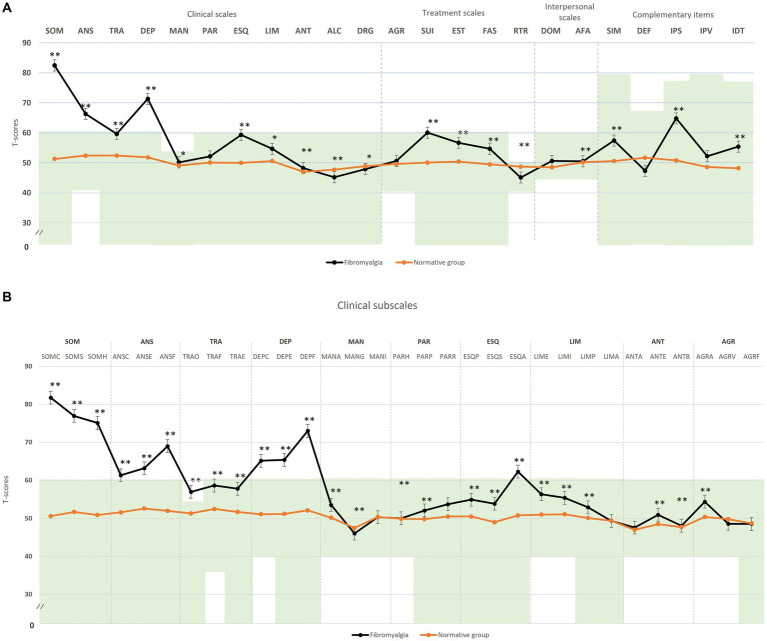
Comparison of means of the psychopathological profile by the Personality Assessment Inventory (PAI) between FM patients with respect to the normative group. **(A)** Clinical scales; **(B)** clinical subscales. The green zone indicates the ranges of normality, according to the psychometric criteria of the PAI. SOM, somatic complaints; ANS, Anxiety; TRA, Disorders related to anxiety; DEP, Depression; MAN, Mania; PAR, Paranoia; ESQ, Schizophrenia; LIM, Limit traits; ANT, Antisocial traits; ALC, Problems with alcohol; DRG, Problems with drugs; AGR, Aggression, SUI, Suicidal ideation; EST, Stress; FAS, Lack of social support; RTR, Refusal to treatment; DOM, Dominance; AFA, Affability; SIM, Simulation index, DEF, Defensiveness index; IPS, Potential suicide index; IPV, Potential index of violence; IDT, Treatment difficulty index; FM, fibromyalgia group; GC, theoretical control group; **(B)** SOM-C, Conversion; SOM-S, Somatization; SOM-H, Hypochondria; ANS-C, Cognitive; ANS-E, Emotional; ANS-F, Physiological; TRA-O, Obsessive-compulsive; TRA-F, Phobias; TRA-E, Posttraumatic stress; DEP-C, Cognitive; DEP-E, Emotional; DEP-F, Physiological; MAN-A, Activity level; MAN-G, Grandeur; MAN-I, Irritability; PAR-H, Hypervigilance; PAR-P, Persecution; PAR-R, Resentment; ESQ-P, Experiences psychotics; ESQ-S, Social indifference; ESQ-A, Alteration of the thought; LIM-E, Emotional instability; LIM-I, Alteration of identity; LIM-P, Problematic interpersonal relationships; LIM-A, Self-aggression; ANT-A, Antisocial behaviors; ANT-E, Egocentrism; ANT-B, Search for sensations; AGR-A, Attitude aggressive; AGR-V, Verbal aggression; AGR-F, Physical assaults; *statistically significant differences between groups; *p < 0.05; **p < 0.01.

Personality assessment inventory (PAI) (Morey, 1991), administered in its Spanish version (Burneo-Garcés et al., 2020), is a widely used, self-administered, 344-item multidimensional tool. It assesses various psychopathological features and personality disorders, providing valuable information for clinical decision-making, treatment planning, and evaluation (Morey, 1991). The PAI comprises 27 scales: four validity scales (infrequency, inconsistency, negative impression management, and positive impression management), five complementary validity scales, 11 clinical scales, five treatment consideration scales, and two interpersonal scales (Figure 1). Additionally, the PAI includes 30 conceptually driven clinical subscales. The combination of clinical and personality scales and subscales can identify various psychopathological profiles, including 15 clinical syndromes and 11 personality disorders (Wolfe et al., 2010). Participants rated the 344 items on a Likert-type scale ranging from 1 (totally false, not at all true) to 4 (very true). Raw scores on all PAI scales and subscales were converted to T-scores (M = 50, SD = 10) based on Spanish norms. Generically, a T-score > 61 suggests a moderate to marked tendency of a prominent psychopathological trait, following the inventory’s usage guidelines (Karlin et al., 2005; Burneo-Garcés et al., 2020; Morey, 1991). However, it is worth noting that some scales may have different cut-off points to be psychometrically meaningful, as indicated in the PAI implementation and interpretation manual (Ortíz-Tallo et al., 2012; Morey and Alarcón, 2013). The PAI has demonstrated suitable reliability and validity in assessing personality and psychopathology in normative, college, and clinical samples (Burneo-Garcés et al., 2020). The internal consistency reliability coefficients (Cronbach’s alpha) (median alpha of 0.80) and interitem correlations for the PAI full scales and subscales (M = 0.24; M = 0.48–0.71, respectively) are acceptable in chronic pain patients, similar to those reported by Morey (1991) and Karlin et al. (2005). Thus, the PAI retains crucial psychometric properties when applied to chronic pain settings (Karlin et al., 2005).

Fibromyalgia impact questionnaire (FIQ) (Burckhardt et al., 1991), administered in its Spanish version (Rivera and González, 2004), is a self-administered multidimensional tool measuring the impact of FM on functional capacity and quality of life. It comprises 10 items referencing the week preceding completion. The FIQ assesses the disease’s influence on physical capacity, the ability to work, and, for those employed, it is impact on job performance, alongside quality of life. It also includes subjective items related to rheumatic symptoms (pain, fatigue, tiredness, and stiffness) and emotional state (anxiety and depression). Scores range from 0 to 100, with higher values indicating greater impact. The FIQ-Spanish version demonstrates good internal consistency (*α* of 0.81), test–retest reliability after 7 days (significant correlations from 0.52 for fatigue and 0.53 for pain to 0.91 for depression), and validity and sensitivity to change (Monterde et al., 2004). This questionnaire is widely used in psychosocial research.

## 3 Data analysis

We conducted a descriptive analysis of sociodemographic and clinical variables to outline the study sample. IBM SPSS software (Version 21.0, IBM Corp, Armonk, NY) was used for all analyses. The data analysis consisted of three main steps:

*Comparison with normative group:* We compared the FM group’s mean scores on the PAI scales and subscales with those of a normative group using a two-tailed Student *t*-test. To control for multiple comparisons, the Bonferroni method was applied to manage error risk. All reported *p-*values were two-tailed. Effect size was assessed with Cohen’s d (Cohen, 1988), with values of 0.20 for a small effect, 0.50 medium effect, and 0.80 a large effect.

*Defining psychopathological profile:* Abnormal ratings for each psychopathology domain were defined in accordance with the PAI usage guidelines based on validated criteria (Wolfe et al., 2010). Abnormal PAI profiles were established provided that two or more scales or subscales had scores above the cut-off point and/or outside the normal range (T-scores of 40–60) or below T-scores of 40. The diagnosis of residual schizophrenia, based on a single PAI criterion (social indifference), was not considered. To globally quantifying the severity of the PAI-defined clinical and personality profiles, we computed a novel index as [(A + B)/2] considering: (A) the mean of ratings of the scales defining a profile with values above the normal range, and (B) 110 (maximum scale value) minus the mean of the ratings defining a profile in terms of values within the normal range.

*Correlation analysis:* We assessed the relationship between psychopathological disorders, defined by the PAI, and fibromyalgia severity measured by FIQ using two measures: FIQ’s total score (FIQ_T) and a reformulated score (FIQ_C). FIQ_C consider solely rheumatological signs (key symptoms) (Pujol et al., 2019) and emotional symptomatology, in line with Keller et al. (2011). It included items such as clinical pain, fatigue, morning tiredness, stiffness, anxiety, and depressive symptoms. Items related to interference with daily activities and work due to pain were excluded, as 95.6% of patients reported work or activity dysfunction at least 1 day a week during assessment. We conducted a correlation analysis using Pearson’s correlation to examine the link between the functional and symptomatic impact of FM, assessed through FIQ_T and the new FIQ_C measures, and various psychopathological profiles identified by the PAI, which define mental issues in FM.

## 4 Results

*First step of the analysis:* We compared FM patients’ psychopathological profiles to the theoretical mean of PAI using T-scores to identify core clinical features present in this rheumatic disease (Figure 1A). FM patients exhibited significantly higher T-scores in several clinical and treatment scales, with mean scores exceeding the defined cut-off point in five scales: somatic complaints (SOM T M = 82.5, SD = 0.9; d = 3.27), anxiety (ANS T M = 66.3, SD = 11.0; d = 1.66), anxiety disorders (TRA T M = 58.6, SD = 9.2; d = 1.00), affective disturbance or depression (DEP T M = 71.3, SD = 10.6; d = 2.15), and increased suicidal ideation (SUI T M = 60.1, SD = 17.6; d = 0.92). The potential suicide risk (IPS T M = 64.8, SD = 9.8; d = 1.41) was significantly higher but remained within the normal range. Apart from the paranoia and aggression scales, other clinical and treatment scales showed statistically significant differences from the PAI normative group but remained within the normal range. In the complementary items, simulation indicators were also significantly higher in FM but within their clinical normal range (SIM T = 57.4, SD = 12.9; d = −0.72).

Regarding the clinical subscales (Figure 1B), patients with FM exhibited statistically significantly higher T-scores in conversion (SOM-C M = 81.8 SD = 13.9; d = 3.05), somatization (SOM-S M = 76.9, SD = 8.3; d = 2.76), illness-health concern (SOM-H M = 75.1, SD = 10.9; d = 2.49), cognitive anxiety (ANS-C M = 61.4, SD = 12.3; d = 1.13), emotional anxiety (ANS-E M = 63.2, SD = 9.5; d = 1.37), physiological anxiety (ANS-F M = 69.0, SD = 11.7; d = 1.91), cognitive depression (DEP-C M = 65.2, SD = 12.5; d = 1.50), emotional depression (DEP-E M = 65.4, SD = 11.8; d = 1.52), physiological depression (DEP-F M = 73.0, SD = 8.3; d = 2.37), and alteration of thought (ESQ-A M = 62.3, SD = 9.9; d = 1.24). Additionally, the level of obsessive symptomatology was significantly higher in these patients (TRA-O M = 56.9, SD = 10.4; d = 0.70), as per the specific PAI criterion for this clinical subscale. While the profile defined by PAI for the remaining clinical subscales in FM patients falls within the normal range, statistically significant differences were observed compared to the normative group.

**Figure 2:**
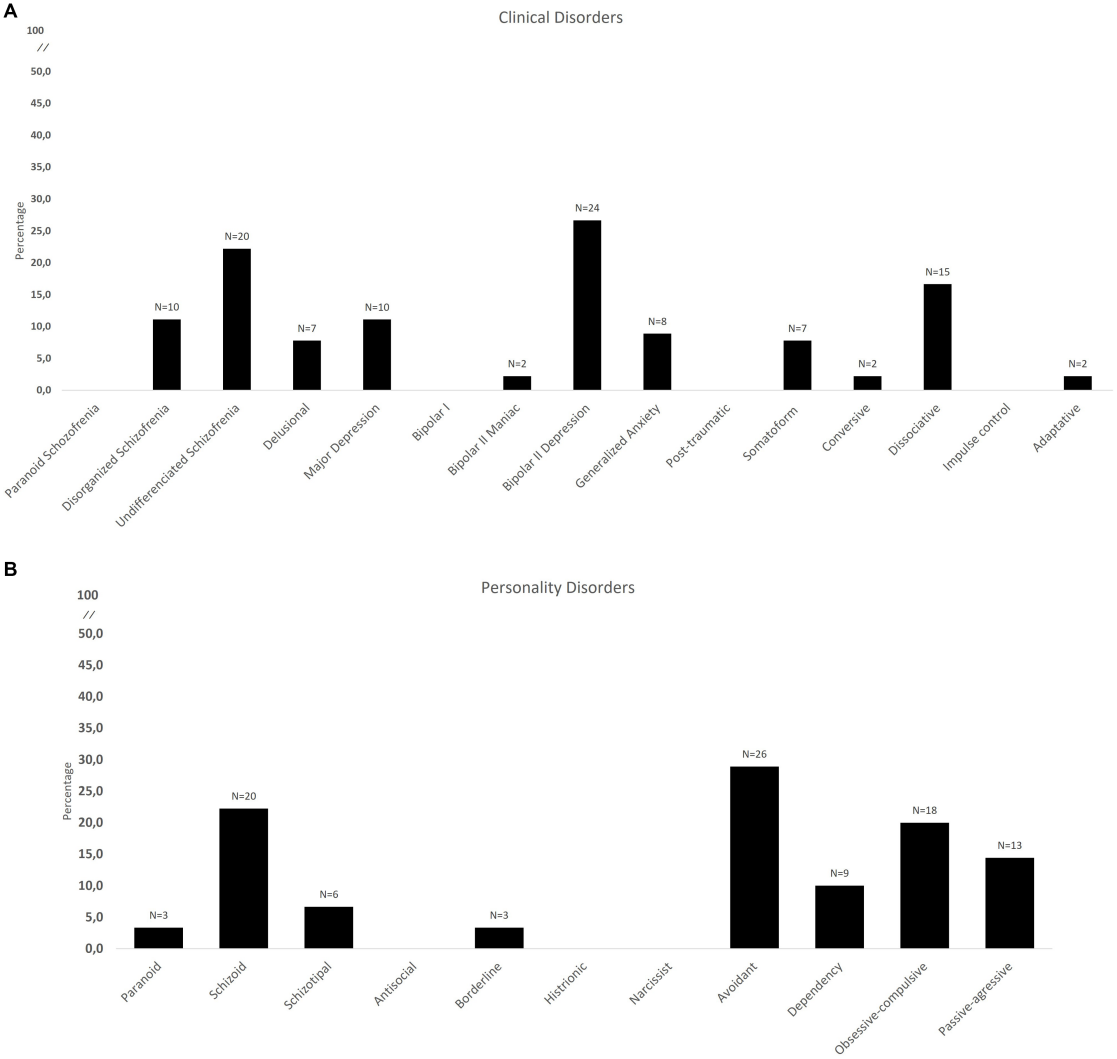
Percentage and number of the presence of the different psychopathological profile defined by PAI criterion; **(A)** major clinical disorders; **(B)** personality disorders.

*Second step of the analysis* (Figures 2A,B):Based on the PAI diagnostic criteria mentioned earlier, 57.7% of FM patients exhibited at least one major mental health disorder. Affective disorders were the most prevalent, accounting for 37.7% of cases (including the depressive factor of bipolar type II scale in 26.6%, major depression in 11.1%, and adaptive disorder in 2.2%). Somatoform spectrum disorders were observed in 9.9% of patients (7.7% somatoform disorder and 2.2% conversion disorder). Dissociative identity disorder, characterized by post-traumatic stress disorder, emotional instability, and negative self-impression, was present in 16.6% of the sample. Psychotic spectrum disorders manifested themselves in 41% (disorganized schizophrenia in 11.1%, undifferentiated schizophrenia in 22.2% and delusional disorder in 7.7%), although the presence of social indifference (social cognition) and medically unexplained somatic experiences in the absence of positive symptomatology is found to play an important role in these disorders. Generalized anxiety disorder was found in 8.8% of patients. Regarding personality disorders, 64.3% of patients exhibited some kind of disorder. Cluster C personality disorders were the most prevalent at 58.8% (including 28.8% avoidant personality disorder, 20.0% obsessive-compulsive personality disorder, and 10.0% dependent personality disorder). Concerning Cluster A personality disorders, 22.2% of cases were schizoid personality disorder.

The psychopathological profile defined by PAI diagnosis criteria (Ortíz-Tallo et al., 2012) predominantly falls into either major depressive disorder (meeting 6 out of 7 PAI criteria) or adaptive disorder with anxious-depressive disturbance (meeting 9 out of 11 PAI criteria). Regarding PDs, the FM group is characterized by obsessive-compulsive personality disorder (meeting 3 out of 3 PAI criteria) and avoidance personality disorder (meeting 3 out of 4 PAI criteria). Concerning psychopathological comorbidity, among patients with established PAI diagnoses, 69.2% met criteria for more than two clinical syndromes. For personality disorders, 48.2% met criteria for more than two PAI diagnosis criteria. The comorbidity between at least one clinical syndrome and a personality disorder was 54.9%.

Third step of the analysis (Tables 2, 3): It is noted a consistent relationship between the severity of psychopathological profile defined by PAI criteria and the impact of the disease, measured by FIQ_T and FIQ_C.

**Table 2:** Pearson’s correlations of FIQ measures with psychopathological and personality profiles defined by PAI criteria.

PAI Measures		FIQ_T	FIQ_C	Pain	Fatigue	Morning tiredness	Stiffness	Anxiety	Depression
Clinical disorders	Paranoid schizophrenia	**0.36 (0.002)**	0.05 (0.680)	0.26 (0.026)	0.29 (0.012)	**0.34 (0.004)**	0.23 (0.055)	0.22 (0.063)	**0.45 (<0.005)**
	Disorganized schizophrenia	0.29 (0.014)	0.22 (0.066)	0.13 (0.280)	0.17 (0.138)	−0.06 (0.624)	−0.06 (0.580)	0.01 (0.930)	0.14 (0.218)
	Undifferentiated schizophrenia	0.22 (0.057)	0.14 (0.244)	0.01 (0.881)	0.09 (0.433)	0.23 (0.048)	0.13 (0.269)	−0.06 (0.592)	0.17 (0.148)
	Delusional	−0.22 (0.066)	−0.19 (0.108)	−0.13 (0.267)	−0.14 (0.226)	−0.16 (0.181)	−0.15 (0.205)	−0.09 (0.419)	−0.14 (0.239)
	Major depression	**0.36 (0.002)**	**0.37 (0.002)**	0.02 (0.871)	0.18 (0.118)	**0.33 (0.005)**	0.23 (0.055)	0.22 (0.063)	**0.45 (<0.005)**
	Bipolar I	−0.10 (0.381)	−0.06 (0.593)	0.01 (0.875)	−0.14 (0.219)	−0.10 (0.391)	−0.05 (0.637)	0.04 (0.702)	−0.06 (0.571)
	Bipolar II maniac	−0.21 (0.069)	−0.20 (0.097)	−0.11 (0.346)	−0.16 (0.170)	**−0.31 (0.009)**	−0.16 (0.168)	0.06 (0.624)	−0.19 (0.114)
	Bipolar II depressive	**0.50 (<0.005)**	**0.49 (0.014)**	0.18 (0.118)	0.22 (0.059)	**0.41 (<0.005)**	**0.30 (0.011)**	**0.32 (0.006)**	**0.52 (<0.005)**
	General anxiety	−0.24 (0.041)	−0.29 (0.014)	−0.12 (0.300)	−0.12 (0.312)	−0.23 (0.048)	−0.24 (0.040)	−0.09 (0.425)	**−0.34 (0.004)**
	PTSD	−0.17 (0.150)	−0.16 (0.173)	−0.12 (0.323)	0.01 (0.936)	−0.06 (0.622)	−0.17 (0.159)	−0.12 (0.293)	−0.16 (0.177)
	Somatic	**0.40 (0.001)**	**0.42 (<0.005)**	0.13 (0.264)	0.22 (0.057)	**0.33 (0.004)**	0.23 (0.051)	**0.32 (0.006)**	**0.43 (<0.005)**
	Conversive	0.03 (0.748)	−0.01 (0.915)	**0.30 (0.010)**	−0.25 (0.030)	0.03 (0.762)	−0.03 (0.803)	−0.13 (0.269)	−0.21 (0.075)
	Dissociative identity	**0.32 (0.006)**	**0.33 (0.005)**	0.13 (0.273)	0.17 (0.145)	0.20 (0.085)	0.21 (0.069)	0.26 (0.027)	**0.32 (0.006)**
	Impulsive control	**0.35 (0.002)**	**0.30 (0.011)**	0.27 (0.020)	**0.46 (<0.005)**	**0.38 (0.001)**	0.16 (0.184)	0.03 (0.774)	0.15 (0.192)
	Adaptative	**0.38 (0.001)**	**0.34 (0.004)**	0.26 (0.026)	0.29 (0.012)	**0.34 (0.004)**	0.18 (0.128)	0.15 (0.196)	0.25 (0.030)
Personality disorders									
	Paranoid	0.04 (0.736)	0.00 (0.973)	−0.13 (0.276)	−0.09 (0.416)	0.04 (0.705)	0.02 (0.849)	−0.04 (0.708)	0.12 (0.309)
	Schizophrenia	0.03 (0.547)	0.02 (0.854)	−0.10 (0.395)	0.02 (0.880)	0.11 (0.327)	0.06 (0.573)	−0.10 (0.388)	0.07 (0.542)
	Schizoid	0.12 (0.296)	0.08 (0.462)	−0.08 (0.482)	−0.02 (0.844)	0.11 (0.341)	0.08 (0.482)	−0.02 (0.812)	0.21 (0.070)
	Antisocial	−0.09 (0.450)	−0.13 (0.282)	0.11 (0.349)	−0.14 (0.239)	−0.10 (0.387)	−0.03 (0.793)	−0.11 (0.332)	−0.13 (0.274)
	Borderline	0.26 (0.030)	**0.32 (0.006)**	−0.03 (0.806)	0.19 (0.111)	0.16 (0.186)	0.15 (0.196)	**0.35 (0.003)**	**0.40 (0.001)**
	Histrionic	0.27 (0.021)	0.23 (0.048)	**0.36 (0.002)**	**0.46 (<0.005)**	0.28 (0.016)	0.11 (0.360)	0.00 (0.987)	0.03 (0.790)
	Narcissist	−0.02 (0.840)	−0.01 (0.883)	0.15 (0.199)	0.21 (0.069)	0.02 (0.837)	−0.03 (0.769)	−0.09 (0.456)	−0.15 (0.192)
	Avoidance	0.20 (0.096)	0.18 (0.126)	0.10 (0.376)	0.16 (0.183)	0.16 (0.167)	0.07 (0.564)	0.01 (0.947)	0.27 (0.023)
	Dependency	0.15 (0.208)	0.17 (0.146)	0.20 (0.088)	**0.32 (0.007)**	0.15 (0.191)	0.04 (0.739)	0.13 (0.271)	0.02 (0.822)
	Obsessive-compulsive	−0.08 (0.560)	−0.14 (0.317)	−0.03 (0.817)	−0.03 (0.818)	−0.06 (0.657)	−0.04 (0.761)	−0.18 (0.204)	−0.19 (0.182)
	Passive-aggressive	0.13 (0.252)	0.14 (0.239)	−0.01 (0.932)	0.02 (0.820)	0.08 (0.495)	0.01 (0.925)	0.15 (0.204)	0.24 (0.043)

FIQ_T, Fibromyalgia impact questionnaire; FIQ_C, Fibromyalgia impact questionnaire corrected; PTSD, Posttraumatic stress disorder. In bold: *r* > 0.30 (medium/large effect).

**Table 3:** Pearson’s correlations of FIQ significantly measures with psychopathological and personality profiles defined by PAI criteria.

PAI measures		FIQ_T	FIQ_C	Pain	Fatigue	Morning tiredness	Stiffness	Anxiety	Depression
Clinical disorders	Paranoid Schizophrenia	0.36 (0.002)	–	–	–	0.34 (0.004)	–	–	0.45 (<0.005)
	Major Depression	0.36 (0.002)	0.37 (0.002)	–	–	0.33 (0.005)	–	–	0.45 (<0.005)
	Bipolar II Maniac	–	–	–	–	−0.31 (0.009)	–	–	–
	Bipolar II Depressive	0.50 (<0.005)	0.49 (0.014)	–	–	0.41 (<0.005)	0.30 (0.011)	0.32 (0.006)	0.52 (<0.005)
	General Anxiety	–	–	–	–	–	–	–	−0.34 (0.004)
	Somatic	0.40 (0.001)	0.42 (<0.005)	–	–	0.33 (0.004)	-	0.32 (0.006)	0.43 (<0.005)
	Conversive	–	–	0.30 (0.010)	–	–	–	–	–
	Dissociative Identity	0.32 (0.006)	0.33 (0.005)	–	–	–	–	–	0.32 (0.006)
	Impulsive Control	0.35 (0.002)	0.30 (0.011)	–	0.46 (<0.005)	0.38 (0.001)	–	–	–
	Adaptative	0.38 (0.001)	0.34 (0.004)	–	-	0.34 (0.004)	–	–	–
Personality disorders									
	Borderline	–	0.32 (0.006)	–	–	–	–	0.35 (0.003)	0.40 (0.001)
	Histrionic	–	–	0.36 (0.002)	0.46 (<0.005)	–	–	–	–
	Dependency	–	–	–	0.32 (0.007)	–	–	–	–

FIQ_T, Fibromyalgia impact questionnaire; FIQ_C, Fibromyalgia impact questionnaire corrected.

It is noteworthy that morning tiredness, a physical fibromyalgia symptom (Table 2), showed a significant association with 9 PAI psychopathological profiles, including paranoid schizophrenia, undifferentiated schizophrenia, major depression, bipolar II-depression, somatic disorder, impulse control disorder, adaptive disorder, manic bipolar II-disorder and generalized anxiety. The correlation coefficients ranging from 0.31 to 0.41 (in absolute value) (*p* < 0.048). Pain and fatigue, two somatic symptoms, were only correlated with 4 PAI psychopathological profiles, including paranoid schizophrenia, conversion disorder, impulse control disorder, and adaptive disorder, with Pearson correlations ranging from 0.25 to 0.46 (in absolute value) (*p* < 0.030). Depression, among the emotional items of FIQ_T and FIQ_C, is linked to 7 PAI psychopathological profiles, including paranoid schizophrenia, major depression, bipolar II-depression, somatic disorder, dissociative disorder, adaptive disorder, and generalized anxiety. These correlations range 0.34–0.52 (in absolute value) (*p* ≤ 0.030). In contrast, anxiety levels in FIQ_T and FIQ_C are only related to 3 PAI psychopathological profiles, specifically bipolar II-depression, somatic disorder, and dissociative identity, with Pearson correlations of 0.26–0.32 (in absolute value) (*p* < 0.027).

Regarding personality disorders (Table 3), a significant relationship is observed between 3 of the 4 somatic symptoms of FM according to the FIQ (pain, fatigue, and morning tiredness) and histrionic personality measures, with correlation values 0.28–0.42 (*p* ≤ 016). Specifically, only fatigue shows a significant association with dependency personality measures (*r* = 0.32; *p* = 0.007). The emotional symptoms of the FIQ are significantly related to borderline personality measures (*r* = 0.35; *p* = 0.003 for anxiety and *r* = 0.40; *p* = 0.001 for depression). Only depression is significantly related to Cluster C personality disorders, specifically avoidant personality measures (*r* = 0.27; *p* = 0.023) and passive-aggressive personality measures (*r* = 0.24; *p* = 0.043).

## 5 Discussion

The study findings reveal that patients with FM predominantly exhibit a psychopathological profile compatible with an affective disorder, along with a comorbid Cluster C personality disorder (anxious type), based on the PAI diagnosis criteria. The predominant psychopathological profile more closely related to the overall impact of the disease’s severity involved an affective disorder, hypervigilance, derealization symptoms, and somatization, often accompanied by a Cluster B personality disorder (emotional instability). However, when focusing solely on key rheumatological and emotional symptoms, the severity of FM is associated with a psychopathological profile characterized by affective and somatic disorders, often comorbid with a Cluster B personality disorder. Therefore, the psychopathological expression may vary among patients depending on their predominant rheumatological symptomatology. For instance, core symptoms such as fatigue and pain are linked to higher levels of suspicion, hypervigilance, impulsivity, and maladaptive reactions; morning tiredness is associated with anxious-depressive symptoms; and stiffness exhibits a selective pattern of anxious-depressive relationship. Notably, greater rheumatological or physical symptomatology is related to an unstable and dependent personality, whereas increased emotional symptomatology (anxious-depressive) is related to a personality characterized by avoidance, borderline traits, and passive-aggressive tendencies.

Our study revealed a notable prevalence of depressive affective disorders within the sample, with either major depression (11.1%) or bipolar II-depression factor (26.6%) being the most prevalent. This emphasizes the dysphoric aspect of FM, aligning with existing literature (García-Fontanals et al., 2017; Gálvez-Sánchez et al., 2019). Major depression was also found alongside generalized anxiety disorder, with an 8.8% prevalence. These findings are consistent with previous research using DSM-IV and SCID-I criteria in chronic pain and FM samples, reporting major depression rates of 15–23.3% and persistent depressive disorder rates between 20 and 51.2% (García-Fontanals et al., 2017; Berkol et al., 2017). This psychopathological profile is particularly relevant as we observed that increased affective disturbance correlates with higher levels of emotional symptomatology, stiffness, and morning fatigue, reflecting the disease’s impact. Furthermore, Berkol et al. (2017) and Gyorfi et al. (2022) found a correlation between anxious-depressive states and FM severity, suggesting that these psychological factors can influence dysfunctional pain processing and significantly exacerbate the disease.

Despite a 7.77% prevalence of somatic disorder in the sample, FM should not be classified as such (Berwick et al., 2022). The cardinal physical symptoms of FM, pain, and fatigue, as measured by the FIQ (Berwick et al., 2022), are not associated with somatic disorder according to the PAI. Instead, they relate to anxious-depressive emotional disturbances, as indicated by the FIQ. Conversely, there is a connection between conversion disorder, defined by the PAI, and FM’s characteristic physical symptoms (pain and fatigue). It is important to note that while conversion involves emotionally driven physical manifestations (Ganslev et al., 2020), numerous studies have not considered FM as a conversion disorder (Näring et al., 2007), corroborating this study’s low percentage of patients diagnosed with conversion disorder (2.22%) by PAI. Avishai Cohen and Zerach (2021) also argue that psychological risk factors like higher anxiety temperament or emotional instability in FM can impact the expression of somatic symptoms and pain perception, potentially leading to misdiagnosis as a conversion disorder.

Our study found a notable presence of dissociative identity disorder (16.6%), consistent with other studies (Näring et al., 2007; Duarte et al., 2019) showing increased somatoform dissociation in FM patients compared to controls. Romeo et al. (2022) even suggested that FM patient dysfunctionality can be predicted by factors including depressive symptoms, somatomorphic dissociation, trauma history, and education level. Dissociative identity disorder, akin to psychosomatic disorder, correlates with higher levels of anxious-depressive emotional disturbance, with symptom severity tied to increased cumulative trauma and somatomorphic or psychomorphic dissociation (Romeo et al., 2022). Patients clinically diagnosed with FM face significant challenges in their social and personal lives, often struggling to carry out daily and professional tasks (Paxman, 2021). Consequently, it is not surprising that these patients may experience feelings of misunderstanding, maladaptation, and social isolation, which can result in significant dysfunctionality. This dysfunctionality might resemble characteristics of schizoid personality disorder, found in 22.2% of our sample.

While our study did not show a diagnosis of impulse control disorder by PAI, other research suggests that higher levels of rheumatological symptoms in FM or increased pain experiences can lead to increased impulsivity or decision-making difficulties (Roman et al., 2018; Yılmaz and Tamam, 2018). This might be due to a potential impairment in response inhibition capacity in patients experiencing more pain and fatigue (Verdejo-García et al., 2009). In this context, we did not find paranoid schizophrenia disorder in our sample, but we found an interesting correlation between this profile psychopathological with fibromyalgia’s impact, especially in morning tiredness and depression symptoms. Predominantly negative symptoms, such as affective flattening, abulia, or alogia, are likely affected by social misunderstanding and maladaptation (Paxman, 2021).

Personality disorders are rooted in innate traits (temperament) and do not cause FM, but they often coexist comorbidly, complicating its clinical course and exacerbating emotional issues. Existing literature shows high comorbidity of PDs in FM, with 64.3% in our study. This aligns with recent studies reporting 10–71.1% of chronic pain patients with Cluster C PDs (Attademo and Bernardini, 2018; Verdejo-García et al., 2009). Contrary, to the usual Cluster B association, specifically histrionic type (Conrad et al., 2013), we found higher prevalence in Cluster C traits-avoidant (28.8%), dependent (10%), and obsessive-compulsive (20%). Personality disorders grouped in Cluster C typically exhibit prominent traits of fear, rigidity, and anxiety. These traits lead to a lack of self-control, anticipatory anxiety, and low tolerance for the unknown, significantly impacting the patient’s functional capacity and potentially exacerbating their medical condition. So, personality and its pathological variations are also associated with the impact of FM and various health-related aspects (Seto et al., 2019; Heyn et al., 2022).

In line with our study, Attademo and Bernardini (2018) found that FM patients with avoidant, obsessive-compulsive, and passive-aggressive personality disorders displayed a greater disease impact with severe affective symptomatology. Esteve et al. (2022) observed that FM patients exhibiting dependent personality disorder presented a greater disease impact with a higher perception of fatigue, where increased autonomy and social support helped to reduce this effect. Furthermore, dependent, schizotypal, schizoid and borderline PD traits could be significant predictors of FM disorder (Romanov et al., 2023). Notably, the prevalence of Cluster B PDS in our sample is anecdotal, and only 3.33% of cases are compatible with borderline personality disorder, but Cluster B traits were linked to more somatic symptoms, emotional disturbances, and greater pain and fatigue perception, as found in recent articles (Gumà-Uriel et al., 2016; Johnson et al., 2020; Sadr et al., 2023).

The study has limitations: (1) a relatively small sample size, though highly selective and homogeneous group, who have long-standing fibromyalgia; (2) strict PAI criteria might yield false negatives in defining the psychopathological profile in FM; (3) there is a more updated and revised version of the FIQ available for use than the one used in this study; (4) the absence of a control group (such as one comprising individuals with another chronic illness known to involve fewer psychological symptoms); (5) the reliance on self-reported data; (6) the focus on symptom severity solely over the past week. Future research could explore PAI-based psychopathological profiles in FM subgroups categorized by FIQ global severity and how these profiles can exacerbate or arbitrate the symptoms of FM as assessed by the FIQ.

In conclusion, we propose that the PAI can be a suitable tool for establishing a psychopathological profile in clinically diagnosed FM patients, as part of a multidimensional psychological assessment, psychopathological factors may modulate pain perception and influence the clinical course of FM. This profile is mainly characterized by an affective disorder, accompanied by a comorbid Cluster C personality disorder (anxious), which may intensify illness’s impact. Therefore, FM severity is not solely determined by fatigue or pain but also by emotional factors and personality traits (Vallejo et al., 2021). These findings highlight the intricate relationship between psychopathological profiles, personality traits, and FM’s impact on patients, can benefit from this thorough evaluation and find better-suited treatments according to their psychological profile, avoiding years of trials and frustrations. Future research with larger samples and longitudinal designs can further elucidate FM’s underlying mechanisms and aid in developing more effective treatments.

## Data Availability

The original contributions presented in the study are included in the article/supplementary material, further inquiries can be directed to the corresponding authors.
